# Role of Valsalva maneuver electrocardiography in the diagnosis of coronary artery disease in senior adults: a prospective cross-sectional study

**DOI:** 10.3389/fcvm.2026.1767251

**Published:** 2026-03-09

**Authors:** Yefei Zhan, Lingbo Zhou, Chunling Lu, Lu Zhang, Zhen Wang, Fangyuan Guan

**Affiliations:** 1 Department of Coronary Care Unit, Ningbo No. 2 Hospital, Ningbo, China; 2 Department of Health Management, Zhejiang Pharmaceutical University, Ningbo, China; 3 Department of Electrocardiogram, Ningbo No. 2 Hospital, Ningbo, China

**Keywords:** computed tomographic coronaryangiography, coronary artery disease, exercise treadmill test, invasive coronary angiography, Valsalva maneuver

## Abstract

**Background:**

Coronary artery disease (CAD) is the leading cause of death worldwide. The exercise treadmill test (ETT) is widely used for CAD diagnosis but unsuitable for patients who are elderly, frail, or have mobility issues. This study examined whether electrocardiogram monitoring during the Valsalva maneuver, known as Valsalva maneuver electrocardiography (VM-ECG), is a potential screening instrument for CAD in these patients.

**Methods:**

This prospective cross-sectional study enrolled 106 elderly patients aged 60–75 years. All participants underwent the ETT, VM-ECG, and coronary imaging (invasive coronary angiography or computed tomography coronary angiography). Diagnostic performance metrics, including sensitivity, specificity, and accuracy, were calculated for VM-ECG. Adverse reactions during ETT and VM-ECG were recorded and compared. Patients were categorized into negative, equivocal, and positive groups based on VM-ECG results. Sequential logistic regression analysis was performed to identify factors that independently influenced VM-ECG outcomes.

**Results:**

VM-ECG demonstrated a maximum sensitivity of 71.4%, specificity of 76.5%, and accuracy of 73.9% for CAD diagnosis. The incidence of adverse reactions was significantly lower during VM-ECG than the ETT (2.8% vs. 20.8%, *P* < 0.0001). Sequential logistic regression analysis identified the maximum heart rate during the expiratory phase (odds ratio = 1.118, 95% confidence interval: 1.046–1.211, *P* = 0.002) as factor independently influencing VM-ECG outcomes.

**Conclusions:**

The VM-ECG exhibits potential as a screening tool for CAD in elderly populations, comparable to the ETT. The observation that the maximum heart rate during the expiratory phase correlates with an increased positivity rate on VM-ECG indicates its potential utility as a critical diagnostic parameter. Further research is necessary to substantiate its long-term diagnostic accuracy, cost-effectiveness, and applicability in real-world settings.

## 1 Introduction

Cardiovascular diseases, particularly coronary artery disease (CAD), represent the leading cause of mortality worldwide (1, 2). While invasive coronary angiography (ICA) remains the gold standard for CAD diagnosis (3), its invasive nature presents significant limitations. Computed tomography coronary angiography (CTCA) has emerged as a sensitive and widely adopted noninvasive imaging modality (4), but it cannot be used to assess cardiac function during physical exertion. Electrocardiography (ECG) remains a cost-effective, reliable, and essential non-invasive method for diagnosing cardiac abnormalities, including CAD, in modern medical practice (5). The exercise treadmill test (ETT) and other exercise stress ECG methods provide key insights for CAD assessment (6), but such tests cannot be completed by many patients, such as those with visual impairments who struggle with balance, those with weak limb muscles who are unable to walk or run, and elderly patients with a high fall risk. For these patients, we propose ECG conducted as patients perform the Valsalva maneuver during expiration, termed Valsalva maneuver electrocardiography (VM-ECG) (7). The Valsalva maneuver is an isometric exercise that increases oxygen use and myocardial blood supply by raising the heart rate (HR) and blood pressure and expanding coronary arteries (8–10). CAD patients show weakened vascular responses, which are linked to coronary endothelium health (11, 12), and ST-segment changes on ECG suggest that VM-ECG could be effective for diagnosing CAD.

The present study investigated the utility of VM-ECG as a novel method for ECG monitoring during the Valsalva maneuver (6). This study specifically aimed to assess the diagnostic efficacy and safety of the VM-ECG as a potential screening instrument for CAD to the ETT for diagnosing CAD in elderly patients.

## 2 Methods

### 2.1 Study design, patient population and setting

This study was designed as a prospective, observational, cross-sectional investigation evaluating the potential efficacy of VM-ECG for the diagnosis of CAD. Patients were recruited based on the following inclusion criteria: 1) suspected CAD; 2) age 60–75 years; 3) ability to complete three examinations: ETT, VM-ECG, and ICA or CTCA; 4) completion of the ETT and VM-ECG under the supervision of researchers; 5) completion of these three examinations within 3 weeks; and 6) provision of written consent by participants or their legal representatives along with a willingness to comply with the protocol.

Patients were excluded according to the following exclusion criteria: 1) a history of coronary artery stenting or coronary artery bypass grafting (CABG); 2) a history of cardiogenic shock or severe heart failure (NYHA class III or higher); or 3) a history of systemic diseases, such as cancer, parathyroid dysfunction, or psychiatric disorders.

The data collected included demographic variables, lifestyle factors, comorbidities, laboratory test results (blood counts, cardiac biomarkers, and metabolic indicators), and diagnostic test results (ETT, VM-ECG, and ICA/CTCA). Patients were categorized based on VM-ECG findings into the following groups: 1) negative, 2) equivocal, and 3) positive. Comparative analyses of demographic data, biomarker levels, and imaging outcomes were performed across groups.

This study was carried out from January 2022 to October 2024 in the Department of Electrocardiography at Ningbo No. 2 Hospital, located in Zhejiang Province, China, which is a Tertiary A-grade comprehensive teaching hospital with a capacity of more than 2,200 beds. Annually, the hospital handles more than 1.8 million outpatient and emergency visits.

### 2.2 ETT

For ETT performance, this study used a 12-lead ECG instrument (Japan Optoelectronics ECG-1350P) and a tablet exercise tester (DMS300-BTT01/BTR01). The exercise continued until the patient achieved their target HR or until the occurrence of symptoms (angina, dyspnea, fatigue, etc.) or rhythm abnormalities (ventricular tachycardia, etc.). The results were interpreted as follows (13–15):

(A) Positive ETT result according to the following criteria:
  1) ECG changes: horizontal or descending ST-segment depression ≥0.1 mV, lasting ≥2 min (with a further ≥0.1 mV depression in cases with baseline ST depression); horizontal or ascending ST-segment elevation ≥0.2 mV, lasting ≥1 min; ascending ST-segment depression >0.2 mV with ST elevation >0.10 mV in lead augmented voltage, right arm (aVR); and transient tall T waves with inverted T waves in corresponding leads.
  2) Clinical manifestations: typical angina during exercise, with ischemic ST-T changes; blood pressure decrease >10 mmHg during exercise, with systemic reactions (e.g., hypotensive shock); and HR decrease ≥20 bpm during exercise, with signs of myocardial ischemia.
(B) Equivocal positive ETT result according to the following criteria: ST-segment depression ≥0.05 mV but <0.1 mV during/after exercise; U-wave inversion during/after exercise; arrhythmias at low workload [<5 metabolic equivalents of task (METs)], such as ventricular premature contractions, ventricular tachycardia, or atrioventricular block; delayed systolic blood pressure response (e.g., decrease of ≥10 mmHg during exercise); and abnormal HR recovery (≤12 bpm decrease at 2 min post-exercise).

### 2.3 VM-ECG

**Figure 1:**
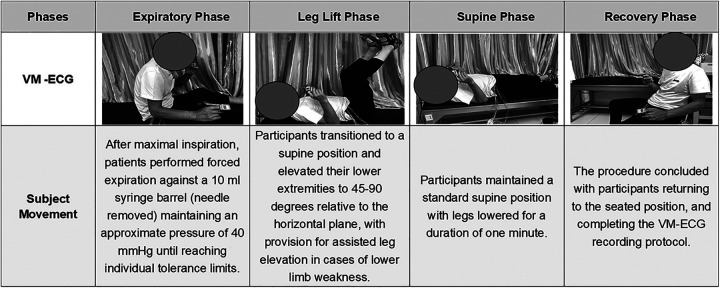
The four-phase process of Valsalva maneuver electrocardiogram (VM-ECG) recording.

VM-ECG refers to electrocardiographic recording as patients perform the Valsalva maneuver, a standardized procedure consisting of four sequential phases: expiratory phase, leg lift phase, supine phase, and recovery phase. According to the protocol used in this study, patients were positioned in a seated posture with continuous ECG monitoring initiated as well as peripheral oxygen saturation (SpO₂) measured prior to maneuver commencement. The detailed movements within the four distinct phases were as follows (Figure 1):

1) Expiratory phase: After maximal inspiration, patients performed forced expiration against a 10-mL syringe barrel (needle removed), maintaining an approximate pressure of 40 mmHg until the individual's tolerance limit was reached. The procedure was discontinued if the patient was unable to continue and voluntarily stopped, developed symptoms such as dizziness or chest pain, or had a SpO₂ level below 92%.
2) Leg lift phase: Participants transitioned to a supine position and elevated their lower extremities to 45°–90° relative to the horizontal plane, with provision for assisted leg elevation for patients with lower limb weakness;
3) Supine phase: Participants maintained a standard supine position with legs lowered for a duration of 1 min;
4) Recovery phase: The procedure concluded with participants returning to the seated position, after which the VM-ECG recording protocol was completed.

The interpretation criteria for VM-ECG results were consistent with those employed for the ETT as described above. This standardized protocol ensured reproducible assessment of cardiovascular responses during the Valsalva maneuver while maintaining patient safety through continuous monitoring and assistance provisions.

### 2.4 ICA

Quantitative coronary angiography was conducted using specialized software (Artis zee III ceiling, Siemens Healthcare, Germany) to measure coronary artery dimensions and calculate the percentage of diameter stenosis for each lesion. Obstructive coronary artery disease was defined by luminal stenosis ≥50%, a validated threshold for increased cardiovascular risk (16). In this study, the ICA result was considered positive if coronary artery stenosis reached or exceeded the 50% threshold.

### 2.5 CTCA

CTCA was performed using a dual-source CT system (SOMATOM Force, Siemens Healthcare, Forchheim, Germany). All acquired CT images were independently evaluated by two board-certified radiologists with expertise in cardiovascular imaging, with consensus in cases of discrepancy reached through discussion. A positive CTCA result was defined as a ≥50% reduction in luminal diameter in at least one major coronary artery segment.

### 2.6 Ethical considerations

The present study followed the tenets of the 1975 Declaration of Helsinki, revised in 1983. The protocol was approved by the Ethics Committee of Ningbo No. 2 Hospital, China (YJ-NBEY-KY-2021-151-01) and registered in the China Medical Research Registration System (MR-33-22-000521). The trial was registered in the Chinese clinical trial registry (ChiCTR2500102401).

### 2.7 Statistical analysis

Statistical analyses were conducted using SPSS 20.0 and MedCalc 16.8. Categorical variables were expressed as frequencies and percentages, and compared using Chi-square or Fisher's exact tests. Continuous variables that followed a normal distribution were presented as mean ± standard deviation, whereas those that followed a non-normal distribution were presented as median [interquartile range (IQR)]. Group comparisons were performed by conducting one-way analysis of variance (ANOVA) for normal data or the Kruskal–Wallis test for non-parametric data. Ordinal logistic regression analysis was conducted to identify factors that influenced the VM-ECG results, with a two-tailed *P*-value <0.05 indicating significance.

The diagnostic performance of VM-ECG was assessed in comparison with imaging, with results classified as true-positive, true-negative, false-positive, or false-negative. The sensitivity, specificity, positive predictive value (PPV), negative predictive value (NPV), and overall accuracy were calculated using statistical software.

## 3 Results

### 3.1 Clinical characteristics of the study population

**Figure 2:**
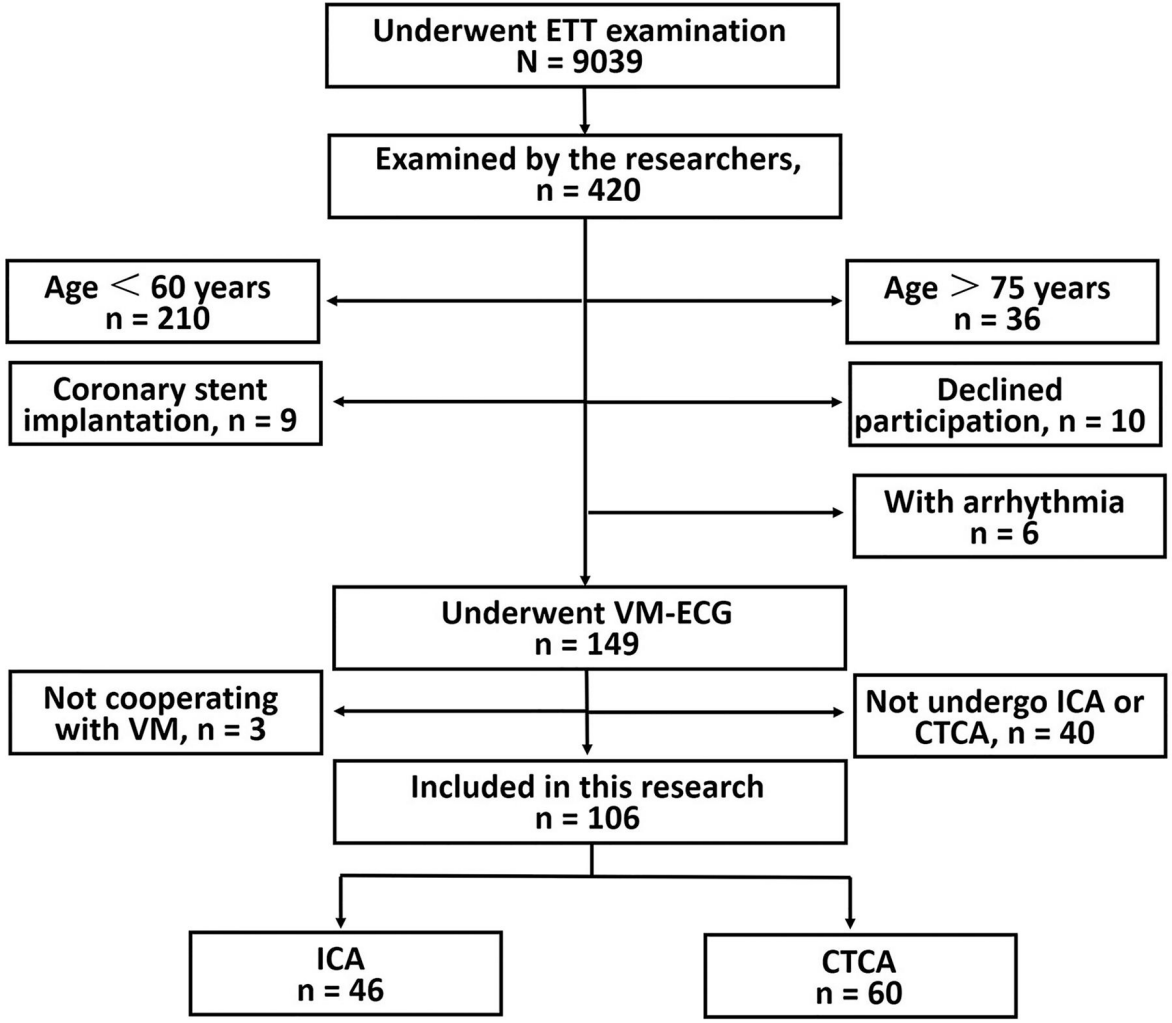
Study flowchart. ETT, exercise treadmill test; VM-ECG, Valsalva maneuver electrocardiogram; ICA, invasive coronary angiography; CTCA, computed tomographic coronary angiography.

Among 9,039 patients who underwent an ETT, 420 were evaluated by the researchers based on a predetermined schedule during the study period. To ensure data quality and patient safety, according to the study criteria patients were excluded for the following reasons: 1) age <60 years (*n* = 210); 2) age >75 years (*n* = 36); 3) prior coronary stenting (*n* = 9); 4) refusal to participate (*n* = 10); 5) significant arrhythmias (*n* = 6); 6) inability to complete vectorcardiography mapping during VM-ECG (*n* = 3); and 7) failure to undergo ICA or CTCA within 3 weeks after the ETT and VM-ECG (*n* = 40). The final cohort included 106 patients (mean age, 66.0 years; 48.6% male) who completed both the ETT and VM-ECG. Of these, 46 (43.4%) underwent ICA, and 60 (56.6%) underwent CTCA for coronary evaluation (Figure 2).

The included patients were divided into three groups with negative, equivocal, and positive VM-ECG results. No significant differences in baseline characteristics, including demographic details, anthropometric factors, comorbidities, or laboratory results (e.g., blood count, lipid panel, glucose, N-terminal pro-brain natriuretic peptide) were observed among the groups (Table 1). In these 106 patients, ultrasound imaging failed to identify significant wall motion abnormalities or complications, including papillary muscle rupture, mural thrombus, or ventricular aneurysm.

**Table 1:** Baseline characteristics of the included patients stratified by VM-ECG results.

Baseline characteristics	Negative VM-ECG (*n* = 36)	Equivocal VM-ECG (*n* = 37)	Positive VM-ECG (*n* = 33)	All (*N* = 106)	*P-*value
Age, year	67.0 (65.0, 70.0)	66.0 (61.0, 68.0)	67.0 (63.0, 69.0)	66.0 (63.0, 69.0)	0.156
Male gender	23 (63.9)	15 (40.5)	13 (39.4)	52 (48.6)	0.066
Body mass index, kg/m^2^	23.3 ± 3.4	24.8 ± 6.0	23.7 ± 2.8	24.0 ± 4.4	0.299
Smoker	14 (38.9)	9 (23.7)	9 (27.3)	32 (29.9)	0.349
Alcohol use	12 (33.3)	8 (21.1)	5 (15.2)	25 (23.4)	0.197
Diabetic mellitus	4 (11.1)	9 (23.7)	5 (15.2)	18 (16.8)	0.341
Hypertension	17 (47.2)	19 (50.0)	21 (63.6)	57 (53.3)	0.352
WBC (*10^3^/µl)	5.5 ± 1.2	5.6 ± 2.2	5.1 ± 1.0	5.4 ± 1.6	0.473
Hemoglobin, g/dl	138.0 ± 11.3	130.0 ± 25.3	126.6 ± 26.7	131.7 ± 22.4	0.118
Platelet (*10^3^/µl)	135 (129, 146)	132 (126, 138)	130 (118, 140)	134 (124, 143)	0.720
D-Dimer, mg/L	125.7 ± 91.2	150.1 ± 79.1	161.1 ± 134.7	146.6 ± 102.9	0.622
CK-MB, U/L	12.0 (11.5, 16.0)	12.0 (9.3, 14.0)	13.0 (11.0, 18.0)	12.0 (11.0, 15.5)	0.120
Serum glucose, mmol/L	5.4 (5.0, 6.3)	5.6 (5.1, 6.9)	5.6 (5.0, 7.7)	5.5 (5.1, 6.6)	0.437
NT-proBNP	100.0 (57.0, 200.0)	147.0 (70.0, 515.0)	183.0 (83.0, 507.3)	145.0 (72.0, 409.5)	0.326
Total cholesterol, mg/dl	177.3 ± 53.9	174.1 ± 53.6	170.9 ± 59.6	174.2 ± 55.1	0.905
LDL cholesterol, mg/dl	107.4 ± 29.8	107.3 ± 33.7	112.6 ± 36.4	109.0 ± 33.1	0.776
HDL cholesterol, mg/dl	54.2 ± 12.9	50.5 ± 11.9	55.5 ± 7.5	53.3 ± 11.2	0.183
Triglyceride, mg/dl	123.0 (88.1, 170.8)	126.6 (104.0, 261.1)	111.5 (93.4, 192.5)	120.4 (94.5, 189.2)	0.667
Echocardiographic data					
Left atrium dimension, mm	35.0 (32.5, 38.0)	35.0 (33.0, 38.0)	33.5 (31.8, 35.0)	36.0 (32.0, 38.0)	0.197
Right ventricular dimension, mm	21.2 ± 2.1	21.3 ± 2.2	21.1 ± 2.6	21.2 ± 2.3	0.985
LV end-diastolic diameter, mm	47.0 (42.5, 50.0)	48.0 (44.0, 50.0)	47.0 (43.8, 50.0)	47.0 (43.8, 50.0)	0.933
LV posterior wall thickness, mm	9.0 (8.0, 10.0)	9.0 (8.0, 10.0)	9.0 (8.0, 9.0)	9.0 (8.0, 10.0)	0.447
Interventricular septal thickness, mm	9.0 (9.0, 10.0)	9.0 (8.0, 10.0)	9.0 (8.0, 10.0)	9.0 (8.0, 10.0)	0.166
LV ejection fraction, %	67.9 ± 4.9	67.1 ± 4.6	67.1 ± 4.9	67.4 ± 4.8	0.720

Values are presented as mean ± SD or median (interquartile range) or number (%).

VM-ECG, Valsalva maneuver electrocardiogram; WBC, white blood cell; CK-MB, creatine kinase MB isoenzyme; NT-proBNP, N-terminal pro-B-type natriuretic peptide; LDL, low-density lipoprotein; HDL, high-density lipoprotein; LV, left ventricular.

### 3.2 VM-ECG diagnosis of CAD

The results for the diagnostic performance of VM-ECG for CAD, assessed compared with ICA and/or CTCA, are summarized in Table 2. When excluding the equivocal VM-ECG group (*n* = 69), VM-ECG demonstrated moderate diagnostic accuracy, with the area under the curve (AUC) values ranging from 0.726 to 0.767, sensitivities from 68.8% to 73.7%, specificities from 71.4% to 84.6%, and overall accuracies from 72.5% to 75.9%. Including the equivocal group in the negative group (positive vs. equivocal/negative, *n* = 106) resulted in decreased sensitivities (47.8%–51.9%) but higher values for specificity (81.8%–91.3%) and PPV (70.0%–84.6%), with lower AUC (0.668–0.696) and accuracy (68.3%–69.6%) values than when excluding equivocal results. Conversely, including the equivocal group in the positive group (equivocal/positive vs. negative, *n* = 106) increased sensitivities (78.3%–81.5%) but substantially decreased specificities (45.5%–47.8%) and PPV (55.0%–60.0%), yielding the lowest AUC (0.630–0.635) and accuracy (61.7%–63.0%) values across all analysis strategies. The highest individual AUC value (0.767) and specificity (91.3%) were observed when using ICA alone as the reference standard and excluding equivocal VM-ECG results.

**Table 2:** Diagnostic performance of VM-ECG in coronary heart disease.

Imaging diagnosis	Positive	Negative	False positive	False negative	AUC	95% CI	Se (%)	Sp (%)	PPV (%)	NPV (%)	Acc. (%)
Exclude VM-ECG Equivocal group (*n* = 69).											
VM-ECG (ICA & CTCA)	25	26	8	10	0.739	0.620–0.838	71.4	76.5	75.8	72.2	73.9
VM-ECG (ICA)	11	11	2	5	0.767	0.574–0.903	68.8	84.6	84.6	68.8	75.9
VM-ECG (CTCA)	14	15	6	5	0.726	0.562–0.845	73.7	71.4	70.0	75.0	72.5
Include the VM-ECG Equivocal group in the negative group (positive vs. equivocal/negative, *n* = 106).											
VM-ECG (ICA & CTCA)	25	48	8	25	0.679	0.581–0.766	50.0	85.7	75.8	64.8	68.9
VM-ECG (ICA)	11	21	2	12	0.696	0.542–0.823	47.8	91.3	84.6	63.6	69.6
VM-ECG (CTCA)	14	27	6	13	0.668	0.535–0.785	51.9	81.8	70.0	67.5	68.3
Include the VM-ECG Equivocal group in the positive group (equivocal/positive vs. negative, *n* = 106).											
VM-ECG (ICA & CTCA)	40	26	30	10	0.632	0.533–0.724	80.0	46.4	57.1	72.2	62.3
VM-ECG (ICA)	18	11	12	5	0.630	0.475–0.768	78.3	47.8	60.0	68.8	63.0
VM-ECG (CTCA)	22	15	18	5	0.635	0.500–0.755	81.5	45.5	55.0	75.0	61.7

VM-ECG, Valsalva maneuver electrocardiogram; AUC, the area under the curve; CI, confidence interval; Se, sensitivity; Sp, specificity; PPV, positive predictive value; NPV, negative predictive value; Acc, accuracy; ICA, invasive coronary angiography; CTCA, computed tomographic coronary angiography.

### 3.3 Component indicators of ETT and VM-ECG in relation to VM-ECG results

Comparative analyses of VM-ECG and ETT parameters across the VM-ECG negative, equivocal, and positive groups revealed significant differences in multiple cardiovascular measures (Table 3). Specifically, the VM-ECG positive group exhibited significantly higher HR values compared to the other groups (Negative + Equivocal): during the expiratory phase [maximum (Max.) HR: *P* < 0.001; minimum (Min.) HR: *P* = 0.001), leg lift phase (Max. HR: *P* = 0.009; Min. HR: *P* = 0.046), supine phase (Max. HR: *P* = 0.027; Min. HR: *P* = 0.005), and recovery phase (Max. HR: *P* = 0.007; Min. HR: *P* = 0.021), and also had a longer expiratory phase duration (*P* = 0.028). No significant differences were observed in blood pressure or SpO₂ parameters. Similarly, for ETT parameters, the positive group demonstrated significantly higher resting HR (*P* = 0.014), peak HR (*P* = 0.049), percentage of peak HR to target HR (*P* = 0.016), and HR values at all recovery time points (1 min: *P* = 0.027; 2 min: *P* = 0.024; 3 min: *P* = 0.009; 4 min: *P* = 0.001; 5 min: *P* = 0.001; 6 min: *P* = 0.001), while exercise duration, target HR, and METs showed no significant group differences.

**Table 3:** Single-factor analysis of VM-ECG and ETT component indicators on VM-ECG results.

Component indicators	Negative VM-ECG (*n* = 36)	Equivocal VM-ECG (*n* = 37)	Positive VM-ECG (*n* = 33)	All (*N* = 106)	*P-*value
VM-ECG					
VM baseline systolic BP, mmHg	135.9 ± 17.3	131.7 ± 13.3	136.5 ± 15.5	134.6 ± 15.4	0.367
VM baseline diastolic BP, mmHg	79.4 ± 10.2	78.0 ± 7.9	79.0 ± 9.1	78.8 ± 9.1	0.796
Duration of expiratory, s	21.5 (16.3, 27.8)	17.0 (15.0, 26.0)	25.0 (20.0, 29.5)	20.5 (16.0, 27.3)	0.028*
Max. HR during expiratory phase, bpm	95.8 ± 14.6	107.2 ± 17.2	113.7 ± 13.3	105.4 ± 16.7	<0.001*
Min. HR during expiratory phase, bpm	78.3 ± 15.9	87.4 ± 13.2	91.4 ± 14.2	85.5 ± 15.3	0.001*
Max. SpO_2_ during expiratory phase, %	98.0 (98.0, 99.0)	98.0 (98.0, 99.0)	98.0 (98.0, 99.0)	98.0 (98.0, 99.0)	0.589
Min. SpO_2_ during expiratory phase, %	98.0 (97.0, 98.0)	98.0 (96.0, 98.0)	98.0 (97.0, 98.0)	98.0 (97.0, 98.0)	0.517
Max. HR during leg lift phase, bpm	104.6 ± 16.6	110.5 ± 17.6	117.1 ± 15.0	110.5 ± 17.1	0.009*
Min. HR during leg lift phase, bpm	73.9 ± 14.4	78.0 ± 13.1	82.3 ± 13.9	78.0 ± 14.1	0.046*
Max. SpO_2_ during leg lift phase, %	98.0 (98.0, 99.0)	98.0 (98.0, 99.0)	98.0 (98.0, 99.0)	98.0 (98.0, 99.0)	0.573
Min. SpO_2_ during leg lift phase, %	97.0 (96.0, 97.8)	97.0 (96.0, 97.5)	97.0 (96.0, 97.5)	97.0 (96.0, 97.0)	0.840
Max. HR during supine phase, bpm	84.8 ± 13.8	89.5 ± 11.9	93.4 ± 13.7	89.1 ± 16.2	0.027*
Min. HR during supine phase, bpm	71.8 ± 12.1	76.8 ± 12.5	81.9 ± 13.1	76.6 ± 13.5	0.005*
Max. SpO_2_ during supine phase, %	98.0 (97.0, 98.0)	98.0 (98.0, 98.0)	98.0 (97.0, 98.0)	98 (97, 98)	0.621
Min. SpO_2_ during supine phase, %	97.0 (96.0, 98.0)	97.0 (96.0, 98.0)	97.0 (96.0, 97.5)	97 (96, 98)	0.535
Max. HR during recovery phase, bpm	87.6 ± 13.4	95.5 ± 12.5	96.6 ± 12.0	93.2 ± 13.2	0.007*
Min. HR during recovery phase, bpm	72.1 ± 12.3	78.5 ± 11.4	79.9 ± 12.9	76.8 ± 12.5	0.021*
Max. SpO_2_ during recovery phase, %	98.0 (98.0, 99.0)	98.0 (98.0, 99.0)	98.0 (97.5, 98.0)	98.0 (98.0, 99.0)	0.923
Min. SpO_2_ during recovery phase, %	97.0 (95.3, 98.0)	97.0 (96.0, 98.0)	97.0 (96.0, 98.0)	97.0 (96.0, 98.0)	0.591
ETT					
Exercise duration, s	312.0 (226.3, 454.8)	283.0 (225.0, 357.5)	289.0 (178.5, 388.5)	290.0 (225.0, 393.0)	0.491
Resting HR, bpm	75.2 ± 15.5	79.5 ± 15.0	85.8 ± 13.8	80.0 ± 15.3	0.014*
Target HR, bpm	130.0 (127.0, 131.0)	130.0 (129.0, 134.5)	130.0 (128.0, 133.0)	130.0 (128.0, 132.3)	0.313
Peak HR, bpm	134.0 (125.3, 140.0)	136.0 (132.0, 141.5)	140.0 (133.0, 150.0)	136.0 (131.0, 142.3)	0.049*
Peak HR/target HR achieved	102.0 (97.3, 107.0)	103.0 (100.0, 108.0)	106.0 (103.0, 113.0)	103.5 (100.0, 109.0)	0.016*
1 min recovery HR, bpm	102.0 (81.0, 114.0)	104.0 (97.5, 114.0)	112.0 (104.5, 124.0)	105.0 (96.0, 115.0)	0.027*
2 min recovery HR, bpm	85.5 ± 19.4	91.0 ± 14.7	96.6 ± 14.8	90.9 ± 16.9	0.024*
3 min recovery HR, bpm	79.6 ± 17.1	84.4 ± 15.6	91.4 ± 13.8	84.9 ± 16.1	0.009*
4 min recovery HR, bpm	78.1 ± 15.6	84.0 ± 11.7	90.5 ± 12.9	84.0 ± 14.3	0.001*
5 min recovery HR, bpm	76.4 ± 15.0	84.2 ± 12.7	88.8 ± 12.9	83.0 ± 14.4	0.001*
6 min recovery HR, bpm	76.0 ± 14.6	83.9 ± 12.7	88.1 ± 13.7	82.5 ± 14.5	0.001*
METs	7.0 (5.7, 9.5)	6.5 (5.3, 7.0)	6.5 (4.7, 7.8)	6.80 (5.30, 7.80)	0.122

Values are presented as mean ± SD or median (interquartile range) or number (%).

VM-ECG, Valsalva maneuver electrocardiogram; ETT, exercise treadmill test; BP, blood pressure; Max, maximum; HR, heart rate; bpm, beats per minute; Min, minimum; SpO_2_, peripheral oxygen saturation; Target HR = 85%*(220-age); Peak HR = Max. HR during ETT; METs, metabolic equivalent of task.

* Statistically significant (*P* < 0.05).

Ordinal logistic regression identified the Max. HR during expiratory phase as an independent predictor of VM-ECG outcomes [odds ratio (OR) = 1.118, 95% confidence interval (CI): 1.046–1.211, *P* = 0.002, Table 4], indicating this parameter may be a critical physiological marker for predicting VM-ECG results. Statistical analysis showed that the AUC for Max. HR during expiratory phase was 0.756 (95% CI: 0.654–0.857), indicating significant diagnostic efficacy (*P* < 0.001). The optimal cutoff value was 108 bpm (sensitivity 75.8%, specificity 69.9%).

**Table 4:** Ordinal logistic regression analysis of VM-ECG and ETT component indicators on VM-ECG results.

Factors	β	SE	Wald χ^2^	OR	95% CI		*P-*value
					Lower	Upper	
Max. HR of expiratory phase, bpm	0.118	0.037	9.991	1.125	1.046	1.211	0.002*

VM-ECG, Valsalva maneuver electrocardiogram; SE, Standard Error; CI, confidence interval; Max, maximum; HR, heart rate; bpm, beats per minute.

* Statistically significant (*P* < 0.05).

### 3.4 Adverse events and discomfort during the ETT and VM-ECG procedures

The safety and patient tolerance of both diagnostic procedures were evaluated. During the ETT, 22 patients (20.8%) reported discomfort, including chest tightness (*n* = 14, 13.2%), chest pain (*n* = 5, 4.7%), dyspnea (*n* = 2, 1.9%), and dizziness (*n* = 1, 0.9%). In contrast, only 3 patients (2.8%) reported mild discomfort during VM-ECG, including chest tightness (*n* = 1, 0.9%), chest pain (*n* = 1, 0.9%), and dizziness (*n* = 1, 0.9%). No cases required early termination of either procedure. The three patients who reported discomfort during VM-ECG had previously experienced discomfort during the ETT, with two showing stenosis on imaging. Overall, the discomfort rate was significantly lower for VM-ECG than for the ETT (2.8% vs. 20.8%, *P* < 0.0001), indicating that VM-ECG has a superior safety profile.

## 4 Discussion

### 4.1 Diagnosis of CAD by VM-ECG

While ETT exhibits variable diagnostic performance for CAD, with sensitivity ranging from 45% to 77% and specificity from 60% to 90% (17–20), the VM-ECG method demonstrates comparable diagnostic performance, offering a complementary option for clinical assessment in older adults.

The VM-ECG methodology employed in this study constitutes a specialized variant of exercise stress electrocardiography. The VM, first documented in 1704, is a technique for vagal stimulation recommended by the American College of Cardiology for cardioversion in cases of stable supraventricular tachycardia (21), with broad multidisciplinary applications (22). This study methodologically advances previous research by tailoring the duration of the VM to align with patient tolerance levels, rather than adhering to a standard 15-second protocol (23–25), enhancing optimization of oxygen consumption and myocardial hypoxia simulation.

Validation through ICA and CTCA has demonstrated the diagnostic efficacy of VM-ECG, with sensitivity values ranging from 68.8% to 73.7% and specificity values between 71.4% and 84.6%, excluding equivocal results. Importantly, VM-ECG provides improved accessibility for elderly individuals by utilizing straightforward techniques such as breath-holding or forced exhalation with assisted positioning. Additionally, it offers enhanced safety, with the incidence of adverse events being approximately 10% of those associated with ETT, without necessitating test interruption.

Moreover, the procedure necessitates only standard ECG equipment, thereby enhancing operational efficiency with a protocol duration of ≤4 min, in contrast to the 10–20 min required for ETT. This efficiency contributes to cost-effectiveness by minimizing resource utilization. In our study, VM-ECG demonstrated a sensitivity of 71.4% and a specificity of 76.5% for the diagnosis of CAD, excluding equivocal results. These metrics are comparable to those of conventional ETT but are somewhat lower than the sensitivity (79%–83%) and specificity (82%–86%) reported for pharmacologic stress echocardiography (26). Although VM-ECG is an emerging technique and its diagnostic accuracy necessitates further validation in larger cohorts, pharmacologic stress echocardiography already benefits from established interpretive criteria. The latter requires ultrasound equipment, intravenous pharmacologic agents, and specialized technicians, resulting in significantly higher costs, particularly when contrast agents are used. In contrast, VM-ECG relies solely on standard ECG equipment and does not require contrast or pharmacological agents, with per-test costs being approximately one-third of those for ETT and one-fifth of those for pharmacologic stress echocardiography.

The streamlined 4-min protocol of VM-ECG facilitates its implementation in primary care settings following basic training and is especially appropriate for elderly patients, as evidenced by an adverse event rate of 2.8% observed in this study. In contrast, pharmacologic stress echocardiography necessitates venous access and advanced imaging support, is associated with more contraindications (such as asthma and conduction blocks), and is less feasible in resource-limited areas. This study, conducted as an exploratory investigation, primarily sought to validate the diagnostic efficacy of VM-ECG for screening CAD.

### 4.2 Predictive value of component indicators in VM-ECG results

This study identified Max. HR during the expiratory phase as an independent predictor of positive VM-ECG outcomes. The Max. HR during the expiratory phase of the positive VM-ECG group (113.7 ± 13.3 bpm) was significantly higher than that of the negative VM-ECG (95.8 ± 14.6 bpm). These findings align with studies showing that CAD patients exhibit more rapid HR elevation during early exercise compared with healthy individuals. The expiratory phase of the VM corresponds to exercise onset, and CAD patients typically show a HR increase (>20–27 bpm) at exercise initiation, which has been found to be associated with a 1.5–2.3-fold higher risk of cardiovascular events, an approximately 2-fold increase in all-cause mortality, and an approximately 2.5-fold higher risk of sudden cardiac death (27, 28). Early exercise-induced tachycardia may serve as a prognostic marker in CAD patients. Gender-specific analyses have revealed that rapid HR elevation during initial exercise is linked to a 1.5-fold higher risk of cardiovascular mortality in females (28) and a 2.3-fold higher risk of cardiovascular mortality in males (29). The association between early exercise tachycardia and higher cardiovascular mortality in CAD patients may result from: 1) autonomic dysfunction: Reduced parasympathetic tone or increased sympathetic activity, impairing heart regulation; 2) myocardial ischemia: Rapid HR raises oxygen demand, potentially causing ischemia and increasing arrhythmia or heart attack risk (27); 3) compromised cardiovascular reserve: Limited heart function may lead to compensatory tachycardia and stress; and 4) structural abnormalities: underlying heart or vascular issues may trigger fatal arrhythmias, raising the mortality risk (28).

### 4.3 Considerations and precautions for VM-ECG implementation

When screening patients to determine the suitability of VM-ECG, cautious consideration is necessary for the following populations: 1) patients with aortic stenosis: The risk of hypotension may be increased due to altered aortic pressure dynamics during performance of the VM (30, 31); 2) patients with recent myocardial infarction: The VM should be avoided within 4–6 weeks post-event to prevent complications; 3) patients with retinopathy: During the VM, there is a risk of peripapillary hemorrhage, particularly in patients with pre-existing fundus lesions (32); 4) patients with exophthalmos: The risk of exacerbating ocular proptosis may be increased during the VM (33); and 5) other patients for whom performance of the VM is not recommended (34–36).

We also propose the following considerations for the implementation of VM-ECG. Patients should avoid stimulants, tobacco, alcohol, and caffeine for a period of 12 h preceding the testing. Although adverse effects are typically mild and transient, encompassing symptoms such as chest discomfort, dizziness, visual disturbances, or temporary amnesia, a comprehensive patient evaluation and preparation are critical for the safe administration of VM-ECG.

### 4.4 Study limitations

This study has several limitations that should be considered: First, as a single-center study, potential selection bias may limit the generalizability of the results. Additionally, with 106 participants, the sample size was relatively small, which was constrained by the single-center design and specific recruitment period. ICA is the gold standard diagnostic methodology for CAD, but this study also used CTCA as a non-invasive alternative. While CTCA is clinically accepted, its use in this research may represent a methodological limitation. While the clinical advantages of VM-ECG were found to be significant in this study, further validation through larger, multicenter studies is necessary. Additionally, while this study provides evidence supporting the value of VM-ECG in elderly populations, it did not include certain high-risk patients who are unable to complete ETT, such as those with NYHA class III–IV heart failure or paralysis. Furthermore, its applicability to different age groups, clinical settings, or broader patient populations remains uncertain. Future research should aim to validate the utility of VM-ECG in populations for whom ETT is absolutely contraindicated.

Despite these limitations, the present study offers valuable preliminary evidence for the diagnostic potential of the novel VM-ECG method for detecting CAD. To the best of our knowledge, this work provides an early clinical assessment of VM-ECG for diagnosing CAD. The findings from this exploratory study should be regarded as hypothesis-generating. Future multicenter prospective cohort studies are essential to confirm and refine our findings, establish precise diagnostic accuracy estimates for VM-ECG, power subgroup analyses adequately, and robustly assess its clinical utility—including comparative evaluations against pharmacologic stress echocardiography—regarding impacts on patient management and outcomes.

## 5 Conclusion

In conclusion, the VM-ECG demonstrates promise as a screening instrument for CAD in elderly populations, exhibiting comparability to the ETT. The finding that the maximum heart rate during the expiratory phase correlates with an increased positivity rate on the VM-ECG suggests its potential utility as a significant diagnostic parameter. Additional research is required to validate its long-term diagnostic accuracy and applicability in practical settings.

## Data Availability

The raw data supporting the conclusions of this article will be made available by the authors, without undue reservation.
